# Self- and interviewer-reported cognitive problems in relation to cognitive decline and dementia: results from two prospective studies

**DOI:** 10.1186/s12916-023-03147-4

**Published:** 2024-01-17

**Authors:** Yuhui Huang, Hui Chen, Mengyan Gao, Xiaozhen Lv, Ting Pang, Shuang Rong, Xin Xu, Changzheng Yuan

**Affiliations:** 1 School of Public Health, the Second Affiliated Hospital, Zhejiang University School of Medicine, Hangzhou, Zhejiang China; 2 Beijing Dementia Key Lab, National Clinical Research Center for Mental Disorders, NHC Key Laboratory of Mental Health (Peking University), Peking University Institute of Mental Health (Sixth Hospital), Beijing, China; 3 Department of Nutrition and Food Hygiene, School of Public Health, Medical College, Wuhan University of Science and Technology, Wuhan, China; 4 Memory, Ageing and Cognition Centre, Department of Pharmacology, Yong Loo Lin School of Medicine, National University of Singapore, Singapore, Singapore; 5 Department of Nutrition, Harvard T.H. Chan School of Public Health, Boston, MA USA

**Keywords:** Self-reported cognitive problems, Interviewer-reported cognitive problems, Cognitive decline, Dementia, Early indicators, Prospective study

## Abstract

**Background:**

Little is known regarding the association of interviewer-reported cognitive problems (ICP) with age-related cognitive decline. We aimed to investigate the independent associations of ICP and the combined associations of ICP and self-reported cognitive problems (SCP) with subsequent cognitive decline and dementia in two prospective cohort studies.

**Methods:**

We included 10,976 Chinese (age = 57.7 ± 8.7) and 40,499 European (age = 64.6 ± 9.4) adults without dementia from the China Health and Retirement Longitudinal Study (CHARLS) and the Survey of Health, Ageing, and Retirement in Europe (SHARE). Self-rated memory (5-point scale) and interviewer-rated frequencies of asking for clarification (6-point scale) were used to define SCP and ICP (dichotomized). Outcomes included objective cognitive test scores (*z*-score transformation) and incident dementia. Generalized estimating equation models were performed to evaluate mean differences in objective cognitive decline. Logistic and Cox regression models were used to estimate the relative risk of dementia. Results from two cohorts were pooled using the random-effects models.

**Results:**

ICP was associated with faster cognitive decline in CHARLS (*β*_CHARLS_ = −0.025 [−0.044, −0.006] *z*-score/year). ICP and SCP were also independently associated with higher risk of dementia in two cohorts (pooled relative risk for SCP = 1.73 [1.30, 2.29]; pooled relative risk for ICP = 1.40 [1.10, 1.79]). In the joint analysis, participants with coexistence of SCP and ICP had the fastest cognitive decline (*β*_CHARLS_ = −0.051 [−0.080, −0.021]; *β*_SHARE_ = −0.024 [−0.043, −0.004]; pooled *β* = −0.035 [−0.061, −0.009] *z*-score/year) and highest risk of dementia (OR_CHARLS_ = 1.77 [1.42, 2.20]; HR_SHARE_ = 2.94 [2.42, 3.59]; pooled relative risk = 2.29 [1.38, 3.77]).

**Conclusions:**

The study suggested that interviewer-reported cognitive problems may be early indicators of cognitive decline and dementia in middle-aged and older adults. A combination of self- and interviewer-reported cognitive problems showed the strongest associations with cognitive decline and dementia.

**Supplementary Information:**

The online version contains supplementary material available at 10.1186/s12916-023-03147-4.

## Background

Dementia, primarily characterized by progressive decline in cognitive and functional abilities, is one of the leading causes of disability and mortality in the elderly worldwide [1, 2]. Due to increasing dementia cases and lack of disease-modifying treatments, early identification of individuals at high risk of developing dementia or at the preclinical stage has received considerable attention [3–5]. Particularly, modifying risk factors and multidomain lifestyle interventions in the early time may help to delay or prevent cognitive decline and dementia for them [4, 6, 7].

Self-reported cognitive problems (SCP), generally known as subjective cognitive decline (SCD) or subjective cognitive complaints (SCC), have been employed as one of the earliest symptomatic manifestations preceding the onset of dementia across different populations [8, 9]. A recent meta-analysis of longitudinal studies suggested that SCP were associated with a 90% increased risk of incident dementia [10]. Although considered as early indicators of dementia, many adults reported SCP probably due to normal aging and affective symptoms (i.e., depression and anxiety), whilst the majority of individuals with SCP wouldn't show progressive cognitive decline [11, 12]. Therefore, simply using the SCP to identify high-risk persons may additionally include many individuals unlikely to develop moderate-to-severe cognitive impairment. Fortunately, informants can notice mild cognitive dysfunction at a slightly more advanced stage than subjects themselves but still before the occurrence of dementia [11]. Previous evidence supported that informant-reported cognitive problems were also indicative of dementia and that the combined associations of self- and informant-reported cognitive problems were stronger comparing to the independent associations of SCP [9, 13, 14]. However, it is of difficulty to ask respective informants of all respondents, especially for those who live alone, in large-scale community studies, whereas obtaining feedback from interviewers is relatively feasible and costs less. Despite large application potentials, it remains unclear whether interviewer-reported cognitive problems (ICP) are independent indicators of age-related cognitive decline and if so, what are the combined associations of SCP and ICP.

To address the research gaps, we aimed to investigate the independent associations of ICP and combined associations of ICP and SCP with subsequent cognitive decline and dementia in two population-based cohort studies.

## Methods

### Study population

This study was conducted among participants from 2 prospective cohorts: the China Health and Retirement Longitudinal Study (CHARLS) and the Survey of Health, Ageing, and Retirement in Europe (SHARE). CHARLS and SHARE were sister cohort studies in the Global Aging Data platform, with similar study designs and objectives. CHARLS was a nationally representative survey of adults 45 years of age or older and their spouses in China [15]. SHARE was a cross-national panel study of people aged 50 and older and their spouses in all 27 countries of the European Union, Switzerland, and Israel [16]. Both cohorts collected sociodemographic, lifestyle, and other health-related information via face-to-face interviews. Detailed descriptions of the cohorts were provided elsewhere [17, 18]. Consistent results from two populations with different cultural and genetic backgrounds could increase the credibility and generalizability of the study findings. CHARLS was ethically approved by the Institutional Review Board at Peking University, and SHARE was ethically approved by the Ethics Committee of the University of Mannheim and the Ethics Council of the Max Planck Society. All participants provided signed informed consent.

In the present study, we used wave 1 (2011) for CHARLS and wave 4 (2011) for SHARE as baseline, when exposures of interest were first available simultaneously, and followed up until wave 4 (2018) for CHARLS and wave 8 (2019) for SHARE. Outcomes included cognitive decline and dementia (detailed inclusion and exclusion criteria for each outcome could be found in the Additional file 1: SMethods). Final population for analyzing cognitive decline comprised 10,976 Chinese in CHARLS and 40,499 Europeans in SHARE (Additional file 1: Figure S1). Final population for analyzing dementia consisted of 8112 Chinese and 44,849 Europeans (Additional file 1: Figure S2).

### Self- and interviewer-reported cognitive problems

Self-rated memory was obtained from the following survey item: “How would you rate your memory at the present time? Would you say it is excellent, very good, good, fair, or poor?”. According to previous studies conducted among Westerners [19, 20], in SHARE, those who reported “fair” or “poor” were treated as having self-reported cognitive problems. Due to cultural differences, Asians may tend to lean toward the negative side and on average be lower than Westerners in self-rating [21, 22]. Hence, in CHARLS, only those who reported “poor” were treated as having SCP.

Interviewer-rated frequency of asking for clarification was derived from the feedback from interviewers after completing the whole investigation. The corresponding item was as follows: “Did the respondent ask for clarification on any questions? Never, almost never, now and then, often, very often, or always?”. In both cohorts, participants whose interviewers reported “often”, “very often”, or “always” were defined as having interviewer-reported cognitive problems. Based on the combined status of SCP and ICP (SCP × ICP), participants were classified into four groups: Non-SCP & Non-ICP, SCP & Non-ICP, Non-SCP & ICP, and SCP & ICP.

### Cognitive function

Cognitive function (including two domains: episodic memory and executive function) was measured biennially, with the same cognitive tests for episodic memory and different cognitive tests for executive function used in two cohorts. In line with previous studies [23–26], tests for episodic memory comprised immediate and delayed word recall for 10 unrelated words. The episodic memory score was calculated as the mean of two recall tasks in CHARLS (ranging from 0 to 10) and the sum of those in SHARE (0-20). In CHARLS, the executive function score included 3 measurements: time orientation (0–5), numerical ability (0–5), and pentagon drawing test (0–1), with a total range of 0 to 11. Differently, in SHARE, the executive function score was the total number of animals named within 1 min in the verbal fluency test. Due to a small number of outliers in this test, scores greater than 45 were re-coded to 45. Therefore, the global cognitive function score was the sum of immediate word recall, delayed word recall, time orientation, numerical ability, and pentagon drawing test in CHARLS, which varied from 0 to 21, whereas that was the sum of immediate word recall, delayed word recall, and verbal fluency test in SHARE, ranging from 0 to 65. To pool results at the same scale across cohorts, cognitive function scores were individually z-transformed in each cohort, with higher scores indicating better cognitive function.

### Dementia

In CHARLS, participants were asked whether they were diagnosed with memory-related diseases that included not only dementia but also brain atrophy and Parkinson’s disease. Therefore, we defined probable dementia with the operational criteria (detailed case definition methods could be found in the Additional file 1: SMethods) used in the English Longitudinal Study of Ageing (ELSA) [27]. Briefly, objective cognitive function tests, informant-reported cognitive status of respondents, functional status, and diagnosis of Alzheimer’s disease, which were only all available in the wave 4 of CHARLS [28], were used together to identify those with probable dementia. Namely, in CHARLS, probable dementia cases were only available at the last follow-up phase (wave 4). Differently, in SHARE, the status of diagnosed dementia was reported by respondents themselves or proxy respondents at each follow-up phase [29].

### Other covariates

The following baseline characteristics were identified as covariates in our study, mainly involving sociodemographic factors, lifestyles, and health conditions. In CHARLS, sociodemographic factors included age (y), gender (male or female), residence (urban or rural), marital status (married or others), education level (illiterate, ≤ primary school, junior high school, or ≥ high school), and household income (in tertiles); lifestyles comprised smoking status (never, former, or current), drinking status (never, former, or current), sleep duration (≤5.0, 5.1–6.0, 6.1–7.0, 7.1–8.0, or >8.0 h), and physical activity level (metabolic equivalent multipliers weighted, in tertiles) [30]; health conditions consisted of BMI (<18.5, 18.5–23.9, 24–27.9, or ≥28 kg/m^2^), depressive symptoms (scores of CESD-10 ≥12, yes or no), restriction on ADL (difficulty in performing one or more activities of daily living, yes or no), and physician-diagnosed history of hypertension, diabetes, heart-related diseases, stroke, and cancer (yes or no) [31].

In SHARE, sociodemographic factors included age, gender, residence, marital status, education level (primary school, middle school, high school, or ≥ college), and household income; lifestyles comprised smoking status, drinking status, vigorous physical activity (>1 per week, 1 per week, or <1 per week), and moderate physical activity (>1 per week, 1 per week, or <1 per week); health conditions consisted of BMI (<18.5, 18.5–24.9, 25–29.9, or ≥30 kg/m^2^), depressive symptoms (scores of EURO-D ≥4, yes or no), restriction on ADL, and physician-diagnosed history of hypertension, diabetes, heart-related diseases, stroke, and cancer [32]; country (e.g., Austria, Germany, Sweden) was also modeled to explain between-country differences [25, 29]. In addition, we included follow-up year and baseline cognitive function score in the multivariate model when analyzing cognitive decline in two cohorts.

### Statistical analyses

Baseline characteristics of the study participants grouped by the combined status of SCP and ICP were compared using the chi-squared test (*χ*^2^) for categorical variables and one-way analysis of variance (ANOVA) for continuous variables. Missing values of categorical variables were imputed to a separate category. Age-specific and gender-specific prevalence of SCP and ICP in 2 cohorts was calculated, respectively.

Generalized estimating equation (GEE) models were used to examine the association between the independent and combined status of SCP and ICP and cognitive decline, with *β* coefficients of the cross-product term of exposure and follow-up year indicating mean differences in rates of cognitive decline across groups. As for dementia, cox proportional hazard regression models were performed to calculate HRs and 95% CIs for relations between the independent and combined status of SCP and ICP and risk of diagnosed dementia in SHARE. Survival time was defined as the time from baseline to the date of incident dementia, loss to follow-up, or study endpoint, whichever came first. Proportional hazard assumption was tested and verified by including a cross-product term with time in the model [33]. In CHARLS, logistic regression models were utilized to estimate ORs of probable dementia and 95% CIs. The final multivariate models adjusted for potential confounders including sociodemographic factors, lifestyles, health conditions (without physician-diagnosed history of chronic conditions), country (only in SHARE), and baseline cognitive function score (only when analyzing cognitive decline). Results from 2 cohorts were then pooled using the inverse variance-weighted random-effects models, which allowed for between-study heterogeneity [34].

We performed an exploratory analysis to investigate whether individuals with both SCP and ICP were at the highest risk of age-related cognitive decline. We conducted several sensitivity analyses to test the robustness of findings: (1) multiple imputation for missing covariates; (2) without excluding those regarded as suspected dementia at baseline; (3) excluding those with the lowest 10 percentages in cognitive function at baseline (only when analyzing cognitive decline); (4) excluding those diagnosed with diseases severely impairing cognition during follow-up (only when analyzing cognitive decline); (5) excluding those with stroke or cancer at baseline; (6) additionally adjusting for physician-diagnosed history of chronic conditions; (7) additionally adjusting for baseline cognitive function (only when analyzing dementia); (8) additionally adjusting for self-rated hearing; (9) excluding those with self-rated poor hearing at baseline. Further, to examine whether the current Asians-specific and Westerners-specific definitions of SCP were appropriate, we exchanged the definitions in 2 cohorts and repeated the analyses. Stratified analyses were performed across subgroups based on major covariates (dichotomized). Effect modification was detected by adding two-way interaction terms of exposure and covariates or three-way interaction terms of exposure, follow-up year, and covariates in the models. All the above statistical analyses were conducted using SAS (version 9.4) and R (version 4.0.5). A two-tailed *P*-value <0.05 was considered to be statistically significant.

## Results

### Participant characteristics

In the population for analyzing cognitive decline, among 10,976 Chinese (57.7 ± 8.7 years) in CHARLS, 49.9% were female and 37.2% had education levels higher than primary school; of 40,499 Europeans (64.6 ± 9.4 years) in SHARE, 57.5% were female and 80.6% had education levels higher than primary school (Table 1). Baseline characteristics of the population for analyzing dementia were similar (data not shown). Participants with both SCP and ICP were more likely to be older; have lower education level, household income, and physical activity level; and have depressive symptoms, restriction on ADL, hypertension, heart-related diseases, stroke, and poorer cognitive function at baseline. Besides, proportions of Non-SCP & Non-ICP decreased by age and those of SCP & ICP increased in 2 cohorts (Additional file 1: Figure S3).

**Table 1:** Baseline characteristics of the study population in CHARLS (*N* = 10,976) and SHARE (*N* = 40,499)

**Characteristic**	**Total**	**Non-SCP & Non-ICP**	**SCP & Non-ICP**	**Non-SCP & ICP**	**SCP & ICP**	***P*-value**
**China Health and Retirement Longitudinal Study (CHARLS)**						
*N*	10,976	7064	2772	628	512	
Age (years)	57.7 ± 8.7	57.1 ± 8.6	57.8 ± 8.4	60.6 ± 9.6	61.9 ± 9.0	<0.001
Female (%)	5480 (49.9)	3192 (45.2)	1534 (55.3)	396 (63.1)	358 (69.9)	<0.001
Rural (%)	6543 (59.6)	3839 (54.3)	1915 (69.1)	412 (65.6)	377 (73.6)	<0.001
Married (%)	9842 (89.7)	6389 (90.4)	2496 (90.0)	534 (85.0)	423 (82.6)	<0.001
Education level (%)						<0.001
Illiterate	2287 (20.8)	1028 (14.6)	730 (26.3)	269 (42.8)	260 (50.8)	
≤ Primary school	4611 (42.0)	2830 (40.1)	1284 (46.3)	280 (44.6)	217 (42.4)	
Junior high school	2557 (23.3)	1941 (27.5)	528 (19.0)	61 (9.7)	27 (5.3)	
≥ High school	1521 (13.9)	1265 (17.9)	230 (8.3)	18 (2.9)	8 (1.6)	
Household income (%)						<0.001
T1	3041 (32.1)	1617 (26.6)	979 (40.5)	232 (43.2)	213 (47.9)	
T2	3193 (33.7)	2046 (33.7)	826 (34.1)	172 (32.0)	149 (33.5)	
T3	3240 (34.2)	2410 (39.7)	614 (25.4)	133 (24.8)	83 (18.7)	
Smoking status (%)						<0.001
Never	6517 (59.4)	4034 (57.1)	1706 (61.5)	418 (66.6)	359 (70.1)	
Former	960 (8.7)	625 (8.8)	241 (8.7)	58 (9.2)	36 (7.0)	
Current	3499 (31.9)	2405 (34.0)	825 (29.8)	152 (24.2)	117 (22.9)	
Drinking status (%)						<0.001
Never	6543 (59.6)	4053 (57.4)	1734 (62.6)	420 (66.9)	336 (65.6)	
Former	865 (7.9)	525 (7.4)	244 (8.8)	45 (7.2)	51 (10.0)	
Current	3568 (32.5)	2486 (35.2)	794 (28.6)	163 (26.0)	125 (24.4)	
BMI (kg/m^2^)						0.001
<18.5	522 (5.5)	293 (4.9)	145 (5.9)	46 (8.2)	38 (8.1)	
18.5–23.9	4956 (52.4)	3094 (51.8)	1304 (53.0)	300 (53.4)	258 (55.2)	
24–27.9	2862 (30.2)	1853 (31.0)	735 (29.9)	153 (27.2)	121 (25.9)	
≥28	1127 (11.9)	736 (12.3)	278 (11.3)	63 (11.2)	50 (10.7)	
Sleep duration (hours)						<0.001
≤5.0	3015 (27.6)	1640 (23.3)	982 (35.6)	193 (30.9)	200 (39.6)	
5.1–6.0	2384 (21.8)	1613 (22.9)	565 (20.5)	115 (18.4)	91 (18.0)	
6.1–7.0	2264 (20.7)	1592 (22.6)	479 (17.4)	108 (17.3)	85 (16.8)	
7.1–8.0	2425 (22.2)	1688 (24.0)	507 (18.4)	149 (23.9)	81 (16.0)	
>8.0	837 (7.7)	508 (7.2)	222 (8.1)	59 (9.5)	48 (9.5)	
Physical activity level (%)						0.016
T1	1544 (33.2)	991 (33.5)	373 (30.5)	95 (36.8)	85 (38.8)	
T2	1553 (33.4)	1013 (34.3)	396 (32.4)	75 (29.1)	69 (31.5)	
T3	1558 (33.5)	952 (32.2)	453 (37.1)	88 (34.1)	65 (29.7)	
Depressive symptoms (%)	2708 (24.7)	1162 (16.4)	1129 (40.7)	172 (27.4)	245 (47.9)	<0.001
Restriction on ADL (%)	1274 (11.6)	626 (8.9)	423 (15.3)	92 (14.6)	133 (26.0)	<0.001
Chronic conditions (%)						
Hypertension	2794 (25.5)	1726 (24.4)	768 (27.7)	145 (23.1)	155 (30.3)	<0.001
Diabetes	701 (6.4)	438 (6.2)	198 (7.1)	35 (5.6)	30 (5.9)	0.262
Heart-related diseases	1365 (12.4)	782 (11.1)	439 (15.8)	66 (10.5)	78 (15.2)	<0.001
Stroke	227 (2.1)	124 (1.8)	64 (2.3)	17 (2.7)	22 (4.3)	<0.001
Cancer	107 (1.0)	54 (0.8)	37 (1.3)	7 (1.1)	9 (1.8)	0.016
Cognitive function (*z*-score)						
Episodic memory	0.00 ± 1.00	0.17 ± 1.00	−0.23 ± 0.91	−0.41 ± 0.98	−0.58 ± 0.83	<0.001
Executive function	0.00 ± 1.00	0.22 ± 0.92	−0.27 ± 1.01	−0.54 ± 0.98	−0.88 ± 0.95	<0.001
Global cognitive function	0.00 ± 1.00	0.24 ± 0.94	−0.31 ± 0.95	−0.60 ± 0.94	−0.94 ± 0.83	<0.001
**Survey of Health, Ageing and Retirement in Europe (SHARE)**						
*N*	40,499	29,177	9657	1021	644	
Age (years)	64.6 ± 9.4	63.6 ± 9.1	66.9 ± 9.5	65.7 ± 10.1	69.4 ± 10.2	<0.001
Female (%)	23,274 (57.5)	16,509 (56.6)	5796 (60.0)	581 (56.9)	388 (60.2)	<0.001
Rural (%)	13,883 (35.8)	9859 (35.3)	3453 (37.1)	362 (37.4)	209 (33.8)	0.006
Married (%)	30,498 (75.3)	22,280 (76.4)	7048 (73.0)	749 (73.4)	421 (65.4)	<0.001
Education level (%)						<0.001
Primary school	7860 (19.4)	4606 (15.8)	2644 (27.4)	314 (30.8)	296 (46.0)	
Middle school	7694 (19.0)	5118 (17.5)	2218 (23.0)	199 (19.5)	159 (24.7)	
High school	16,181 (40.0)	12,265 (42.0)	3443 (35.7)	329 (32.2)	144 (22.4)	
≥ College	8764 (21.6)	7188 (24.6)	1352 (14.0)	179 (17.5)	45 (7.0)	
Household income (%)						<0.001
T1	13,499 (33.3)	8316 (28.5)	4363 (45.2)	434 (42.5)	386 (59.9)	
T2	13,500 (33.3)	9806 (33.6)	3182 (33.0)	331 (32.4)	181 (28.1)	
T3	13,500 (33.3)	11,055 (37.9)	2112 (21.9)	256 (25.1)	77 (12.0)	
Smoking status (%)						<0.001
Never	20,972 (52.5)	14,836 (51.5)	5211 (54.8)	550 (54.6)	375 (59.2)	
Former	11,294 (28.3)	8241 (28.6)	2649 (27.9)	253 (25.1)	151 (23.9)	
Current	7674 (19.2)	5716 (19.9)	1646 (17.3)	205 (20.3)	107 (16.9)	
Drinking status (%)						<0.001
Never	3602 (9.9)	2359 (8.9)	1012 (11.9)	142 (15.6)	89 (15.8)	
Former	3832 (10.5)	2356 (8.9)	1265 (14.9)	107 (11.7)	104 (18.4)	
Current	28,987 (79.6)	21,747 (82.2)	6204 (73.2)	664 (72.7)	372 (65.8)	
BMI (kg/m^2^)						<0.001
<18.5	428 (1.1)	287 (1.0)	113 (1.2)	17 (1.7)	11 (1.8)	
18.5–24.9	13,976 (35.5)	10,364 (36.5)	3094 (33.1)	340 (34.9)	178 (29.1)	
25–29.9	16,307 (41.5)	11,772 (41.5)	3873 (41.4)	395 (40.6)	267 (43.7)	
≥30	8608 (21.9)	5953 (21.0)	2278 (24.3)	222 (22.8)	155 (25.4)	
Vigorous physical activity (%)						<0.001
>1 per week	14,509 (35.8)	11,398 (39.1)	2690 (27.9)	313 (30.7)	108 (16.8)	
1 per week	5917 (14.6)	4519 (15.5)	1189 (12.3)	135 (13.2)	74 (11.5)	
<1 per week	20,073 (49.6)	13,260 (45.4)	5778 (59.8)	573 (56.1)	462 (71.7)	
Moderate physical activity (%)						<0.001
>1 per week	28,763 (71.0)	21,458 (73.5)	6345 (65.7)	632 (61.9)	328 (50.9)	
1 per week	5550 (13.7)	3937 (13.5)	1368 (14.2)	138 (13.5)	107 (16.6)	
<1 per week	6186 (15.3)	3782 (13.0)	1944 (20.1)	251 (24.6)	209 (32.5)	
Depressive symptoms (%)	10,641 (26.3)	6074 (20.8)	3911 (40.5)	289 (28.3)	367 (57.0)	<0.001
Restriction on ADL (%)	3510 (8.7)	1829 (6.3)	1391 (14.4)	122 (11.9)	168 (26.1)	<0.001
Chronic conditions (%)						
Hypertension	17,039 (42.1)	11,503 (39.4)	4744 (49.1)	427 (41.8)	365 (56.7)	<0.001
Diabetes	4861 (12.0)	3143 (10.8)	1482 (15.3)	117 (11.5)	119 (18.5)	<0.001
Heart-related diseases	5792 (14.3)	3511 (12.0)	1963 (20.3)	147 (14.4)	171 (26.6)	<0.001
Stroke	1684 (4.2)	938 (3.2)	659 (6.8)	35 (3.4)	52 (8.1)	<0.001
Cancer	2835 (7.0)	1928 (6.6)	811 (8.4)	52 (5.1)	44 (6.8)	<0.001
Cognitive function (*z*-score)						
Episodic memory	0.00 ± 1.00	0.14 ± 0.98	−0.34 ± 0.96	−0.27 ± 1.02	−0.88 ± 0.90	<0.001
Executive function	0.00 ± 1.00	0.11 ± 1.01	−0.25 ± 0.90	−0.30 ± 0.99	−0.70 ± 0.75	<0.001
Global cognitive function	0.00 ± 1.00	0.14 ± 0.99	−0.33 ± 0.91	−0.34 ± 0.99	−0.88 ± 0.76	<0.001

*Abbreviations: SCP* Self-reported cognitive problems, *ICP* Interviewer-reported cognitive problems, *BMI* Body mass index, *ADL* Activities of daily living, *T* Tertile

Values were presented as mean ± SD for continuous variables and *n* (%) for categorical variables

The one-way analysis of variance and chi-squared test were used to test for differences across groups

### SCP and ICP with cognitive decline

During an average of 5.4 and 5.7 years of follow-up, rates of cognitive decline were −0.039 (−0.044, −0.034) *z*-score/year for Chinese and −0.027 (−0.029, −0.025) *z*-score/year for Europeans. SCP showed no statistical relationships with global cognitive score decline (pooled *β* = −0.003 [−0.008, 0.003]; Additional file 1: Table S1) while SCP were related to faster cognitive decline in the domain of episodic memory in Chinese (*β* = −0.023 [−0.036, −0.010]; data not shown). Chinese with ICP had faster cognitive decline (*β* = −0.025 [−0.044, −0.006]) while the association was not observed among Europeans (*β* = −0.006 [−0.017, 0.006]; pooled *β* = −0.014 [−0.032, 0.005]). When further mutually adjusting for SCP and ICP, results did not materially change. In the joint association analyses, compared to participants without SCP and ICP, those with coexistence of SCP and ICP were associated with faster cognitive decline (*β*_Chinese_ = −0.051 [−0.080, −0.021]; *β*_Europeans_ = −0.024 [−0.043, −0.004]; pooled *β* = −0.035 [−0.061, −0.009] z-score/year) whereas the corresponding differences were non-significant for those with only SCP or ICP (Table 2). Significant relations between coexistence of SCP and ICP with episodic memory-specific and executive function-specific cognitive decline were also found (Additional file 1: Figure S4).

**Table 2:** Combined association of SCP and ICP with cognitive decline among Chinese (*N* = 10,976) and European (*N* = 40,499) middle-aged and older participants

	**Non-SCP & Non-ICP**	**SCP & Non-ICP**	**Non-SCP & ICP**	**SCP & ICP**
**China Health and Retirement Longitudinal Study (CHARLS)**^**a**^				
*N*	7064	2772	628	512
Age-adjusted model	0 (ref.)	−0.003 (−0.015, 0.010)	−0.008 (−0.034, 0.018)	−0.061 (−0.092, −0.031) *
Multivariate model 1 (MV1)	0 (ref.)	−0.003 (−0.015, 0.009)	−0.008 (−0.033, 0.017)	−0.052 (−0.082, −0.023) *
Multivariate model 2 (MV2)	0 (ref.)	−0.003 (−0.015, 0.009)	−0.008 (−0.033, 0.017)	−0.051 (−0.080, −0.021) *
**Survey of Health, Ageing and Retirement in Europe (SHARE)**^**b**^				
*N*	29,177	9657	1021	644
Age-adjusted model	0 (ref.)	−0.001 (−0.005, 0.004)	0.002 (−0.013, 0.016)	−0.028 (−0.048, −0.008) *
Multivariate model 1 (MV1)	0 (ref.)	−0.0003 (−0.005, 0.005)	0.003 (−0.011, 0.017)	−0.024 (−0.044, −0.004) *
Multivariate model 2 (MV2)	0 (ref.)	0.0002 (−0.005, 0.005)	0.004 (−0.011, 0.018)	−0.024 (−0.043, −0.004) *
**Pooled results**^**c**^				
Multivariate model 2 (MV2)	0 (ref.)	−0.0003 (−0.005, 0.004)	0.001 (−0.011, 0.013)	−0.035 (−0.061, −0.009) *

*Abbreviations: SCP* Self-reported cognitive problems, *ICP* Interviewer-reported cognitive problems

Age-adjusted model: adjusted for age, age^2^, and follow-up year

Multivariate model 1 (MV1): CHARLS: further adjusted for gender, residence, marital status, education level, household income, smoking status, drinking status, sleep duration, BMI, depressive symptoms, ADL, and physical activity level; SHARE: further adjusted for gender, residence, marital status, education level, household income, smoking status, drinking status, BMI, depressive symptoms, ADL, vigorous physical activity, moderate physical activity, and country

Multivariate model 2 (MV2): further adjusted for baseline cognitive function score

Estimates of the mean differences in cognitive decline across groups were* β* coefficients of cross-product terms of exposure and follow-up year

^a^During an average of 5.4 years of follow-up, the rate of cognitive decline was −0.039 (−0.044, −0.034) *z*-score/year for Chinese

^b^During an average of 5.7 years of follow-up, the rate of cognitive decline was −0.027 (−0.029, −0.025) *z*-score/year for Europeans

^c^Results from the multivariate model 2 (MV2) were pooled using the random-effects model

^*^P <0.05

### SCP and ICP with dementia

At the 7th year of follow-up in CHARLS, 1120 incident probable dementia cases were identified while a total of 1524 incident diagnosed dementia cases were reported during the follow-up (mean = 6.0 years) in SHARE. In the multivariate models, SCP and ICP were respectively associated with 73% (30%, 129%) and 40% (10%, 79%) increased risk of dementia, with similar results after mutually adjusting for SCP and ICP (Additional file 1: Table S2). Besides, compared to participants without SCP and ICP, Chinese and Europeans with coexistence of SCP and ICP were associated with higher odds (OR = 1.77 [1.42, 2.20]) and hazards (HR = 2.94 [2.42, 3.59]) of dementia respectively (pooled relative risk = 2.29 [1.38, 3.77]; Table 3). Associations for those with only SCP or ICP were attenuated but remained significant, with pooled relative risk ranging from 1.38 to 1.72.

**Table 3:** Combined association of SCP and ICP with dementia among Chinese (*N* = 8112) and European (*N* = 44,849) middle-aged and older participants

	**Non-SCP & Non-ICP**	**SCP & Non-ICP**	**Non-SCP &** **ICP**	**SCP & ICP**
**China Health and Retirement Longitudinal Study (CHARLS): Probable dementia, OR (95% CI)**^**a**^				
*N*	4495	2276	632	709
Cases	425	405	100	190
Age-adjusted model	1 (ref.)	2.03 (1.75, 2.36) *	1.47 (1.15, 1.87) *	2.89 (2.37, 3.53) *
Multivariate model 1 (MV1)	1 (ref.)	1.47 (1.25, 1.72) *	1.18 (0.91, 1.52)	1.77 (1.42, 2.20) *
**Survey of Health, Ageing and Retirement in Europe (SHARE): Diagnosed dementia, HR (95% CI)**^**b**^				
*N*	31,259	11,152	1276	1162
Cases	637	672	64	151
Person-years	190,818	66,394	7424	6142
Incidence rate^d^	333.8	1012.1	862.1	2458.5
Age-adjusted model	1 (ref.)	2.27 (2.03, 2.53) *	1.98 (1.53, 2.56) *	4.27 (3.56, 5.13) *
Multivariate model 1 (MV1)	1 (ref.)	1.99 (1.77, 2.24) *	1.61 (1.24, 2.10) *	2.94 (2.42, 3.59) *
**Pooled results**^**c**^				
Multivariate model 1 (MV1)	1 (ref.)	1.72 (1.28, 2.31) *	1.38 (1.01, 1.88) *	2.29 (1.38, 3.77) *

*Abbreviations: SCP* Self-reported cognitive problems, *ICP* Interviewer-reported cognitive problems

Age-adjusted model: adjusted for age and age^2^

Multivariate model 1 (MV1): CHARLS: further adjusted for gender, residence, marital status, education level, household income, smoking status, drinking status, sleep duration, BMI, depressive symptoms, ADL, and physical activity level; SHARE: further adjusted for gender, residence, marital status, education level, household income, smoking status, drinking status, BMI, depressive symptoms, ADL, vigorous physical activity, moderate physical activity, and country

^a^Odds ratios (OR) were estimated using the logistic regression model

^b^Hazard ratios (HR) were estimated using the cox proportional hazards model

^c^Results from the multivariate model 1 (MV1) were pooled using the random-effects model

^d^Incidence per 100,000 person-years

^*^P <0.05

### Exploratory analysis, subgroup analyses, and sensitivity analyses

**Fig. 1:**
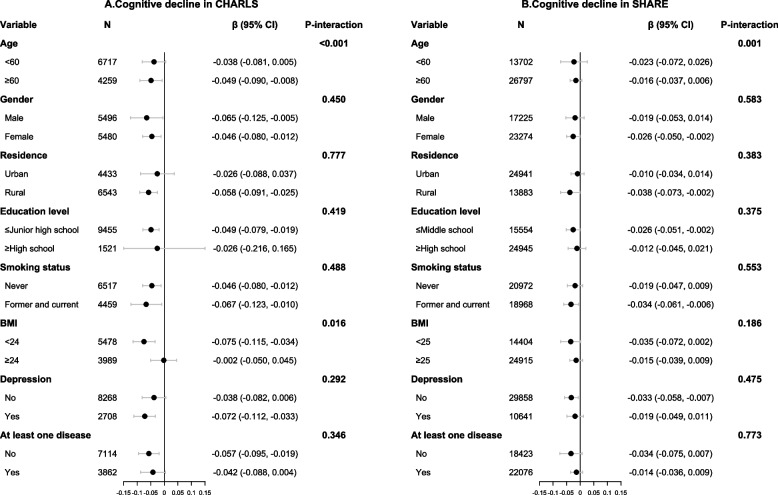
Subgroup analyses for the combined association of SCP and ICP with cognitive decline in CHARLS (**A**) and SHARE (**B**) by comparing SCP & ICP group and Non-SCP & Non-ICP group in the fully adjusted model

**Fig. 2:**
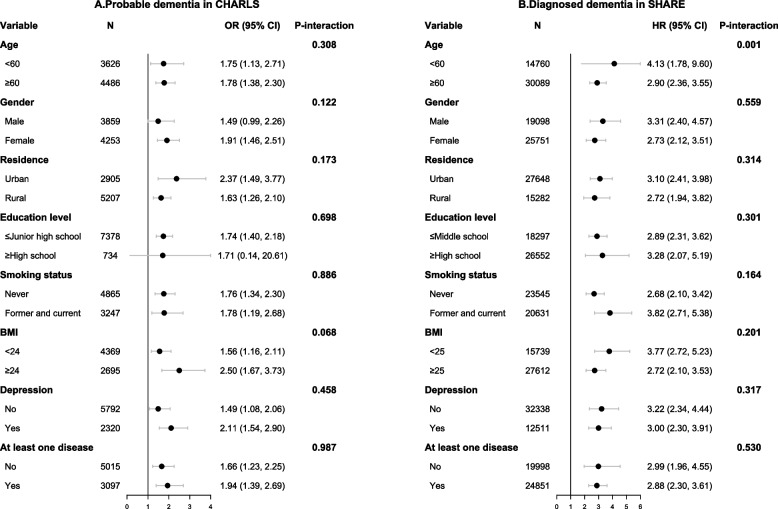
Subgroup analyses for the combined association of SCP and ICP with dementia in CHARLS (**A**) and SHARE (**B**) by comparing SCP & ICP group and Non-SCP & Non-ICP group in the fully adjusted model

In the exploratory analysis, we observed that the SCP & ICP group had the fastest cognitive decline and highest risk of dementia among four groups (Additional file 1: Table S3-S4). In the subgroup analyses, we found no statistical evidence for effect modification by gender, residence, education level, smoking status, depressive symptoms, and history of chronic conditions. However, the combined associations of SCP and ICP with cognitive decline were stronger among those aged ≥60 in CHARLS (*P*-interaction <0.001) whereas opposite effect modification results for cognitive decline and dementia were observed in SHARE (both *P*-interaction = 0.001; Figs. 1 and 2). Additionally, relationships of SCP and ICP with cognitive decline were stronger among those with BMI <24 in CHARLS (*P*-interaction = 0.016). In the sensitivity analyses, results were similar to the main analysis, indicating the robustness of observed associations (Additional file 1: Table S5-S8). When we exchanged the definitions of SCP in 2 cohorts, the corresponding associations were moderately attenuated.

## Discussion

In the two community-based prospective cohort studies of Chinese (7-year follow-up) and European (8-year follow-up) middle-aged and older adults without dementia, we observed independent associations of interviewer-reported cognitive problems with faster cognitive decline and higher risk of incident dementia. Additionally, the strongest associations with age-related cognitive decline were found among participants with the co-existence of self- and interviewer-reported cognitive problems.

In the current study, we observed that SCP were related to faster cognitive decline in the domain of episodic memory in CHARLS. Consistent with our results, one community-based study including 4015 older adults (age = 77.7 ± 7.2 years) found that those with memory complaints had faster decline in the domain of episodic memory (*β* = −0.037 [−0.047, −0.027]) during a mean follow-up of 6 years [35]. Besides, SCP were related to 73% increased risk of developing dementia in our study, which was similar to the pooled results from a meta-analysis (HR = 1.90 [1.52, 2.36]) [10]. Therefore, our findings provided additional evidence to support SCP as indicators of cognitive decline and dementia. In addition, we found that ICP were associated with faster cognitive decline and elevated risk of dementia. Although there is no research exploring whether ICP were related to age-related cognitive decline, several previous studies investigated the associations of informant-reported cognitive problems. For example, one study with 1048 individuals (age = 73.3 ± 7.0 years) from the Alzheimer’s Disease Neuroimaging Initiative showed that worse informant-ECog (Everyday cognition scale) scores were associated with greater decline in multiple neuropsychological tests and increased risk of conversion from mild cognitive impairment (MCI) to dementia [36]. With the large sample size and diverse education levels, these two prospective cohort studies provided strong evidence to support the indicative role of cognitive problems reported by interviewers in age-related cognitive decline.

In the joint association analysis, the coexistence of SCP and ICP was associated with the fastest cognitive decline and highest risk of incident dementia. Similarly, previous studies found that participants with both self- and informant-reported cognitive problems were related to increased risk of cognitive decline and progression from MCI to dementia, with effect estimates greater than those only having one of the two sources of reported cognitive problems [13, 37]. Particularly, one study conducted among community-dwelling older adults (age = 78.7 ± 4.8 years) found that self-reported memory decline may indicate faster cognitive decline in the domain of language while informant-reported memory decline may be indicative of decline in the domain of executive function and memory [38]. Hence, cognitive problems reported by the respondent himself or a third person may represent early signs of future decline in different cognitive domains, which was probably one of the reasons why the coexistence of SCP and ICP showed the strongest relationships with age-related cognitive decline. In addition, compared to informants, obtaining feedback from interviewers may be more feasible and cost less in the community-based studies. Therefore, a combination of self- and interviewer-reported cognitive problems could be employed as a complementary approach to identify those at high risk of age-related cognitive decline, especially in situations when finding appropriate informants was unavailable or difficult.

The present study has a number of strengths. The prospective study design, long-term follow-up, large sample size, inclusion of two populations with different cultural backgrounds and education levels, and careful control of various potential confounders minimized selection bias and reverse causation, thus providing relatively valid estimates of associations. Nevertheless, several limitations should be considered when interpreting the results of our study. The primary limitation is the nature of an observational study design where the observed associations may be impacted by residual and unmeasured confounders. However, we statistically adjusted for a wide range of key risk factors including baseline cognitive function, suggesting that confounding is not a likely explanation for the current findings. Second, the incidence of dementia was measured in an unstandardized way between the two cohorts. However, ways of utilizing the operational criteria and self- or proxy-reported diagnosis to define dementia were both widely used and validated in epidemiological studies [27, 39–41]. More studies with standardized ways to define dementia are warranted to confirm and elucidate the observed findings. Another limitation is the possible reverse causality. We excluded dementia cases occurring within the first 2 years of follow-up when analyzing dementia. Additionally, we repeated the analyses for cognitive decline after excluding those diagnosed with diseases severely impairing cognition during the follow-up and observed similar findings. Fourthly, the use of self-rated current memory performance instead of memory change to define SCP may be subject to underestimation. Nevertheless, such situations potentially included more at-risk persons in the reference group and biased our association estimates toward the null. Finally, some participants may be regarded as having ICP due to language barriers or hearing impairment instead of cognitive problems. However, native speakers were recruited and trained to become interviewers, and feedback from interviewers used to define ICP focused on asking for clarification rather than repetition of questions. We also observed similar results after additionally adjusting for self-rated hearing or excluding those with self-rated poor hearing. More studies with diverse methods to define SCP and ICP are warranted to confirm and elucidate the observed findings.

## Conclusions

Interviewer-reported cognitive problems and the coexistence of self- and interviewer-reported cognitive problems were associated with faster cognitive decline and higher risk of dementia. The study findings suggested that interviewer-reported cognitive problems may be early indicators of age-related cognitive decline in middle-aged and older adults across different populations. A combination of self- and interviewer-reported cognitive problems could be utilized to identify individuals at high risk of developing cognitive decline and dementia, providing an important time window to delay and prevent dementia.

### Supplementary Information

**Additional file 1:** **SMethods, Figures S1-S4, and Tables S1-S8.** **SMethods.** [Supplementary Methods]. **Figure S1.** [Flow chart of participants selection for analyzing cognitive decline]. **Figure S2.** [Flow chart of participants selection for analyzing dementia]. **Figure S3.** [Age-specific and gender-specific prevalence of self-reported cognitive problems (SCP) and interviewer-reported cognitive problems (ICP) in CHARLS (A) and SHARE (B)]. **Table S1.** [Association of SCP and ICP on cognitive decline among Chinese (*N* = 10,976) and European (*N* = 40,499) middle-aged and older participants]. **Figure S4.** [Combined association of SCP and ICP on cognitive decline in the domains (episodic memory and executive function) among Chinese (*N* = 10,976) and European (*N* = 40,499) middle-aged and older participants]. **Table S2.** [Association of SCP and ICP on dementia among Chinese (*N* = 8112) and European (*N* = 44,849) middle-aged and older participants]. **Table S3.** [Combined association of SCP and ICP on cognitive decline among Chinese (*N* = 10,976) and European (*N* = 40,499) middle-aged and older participants (SCP & ICP group as reference)]. **Table S4.** [Combined association of SCP and ICP on dementia among Chinese (*N* = 8112) and European (*N* = 44,849) middle-aged and older participants (SCP & ICP group as reference)]. **Table S5.** [Sensitivity analyses for the combined association of SCP and ICP on cognitive decline in CHARLS]. **Table S6.** [Sensitivity analyses for the combined association of SCP and ICP on cognitive decline in SHARE]. **Table S7.** [Sensitivity analyses for the combined association of SCP and ICP on probable dementia in CHARLS]. **Table S8.** [Sensitivity analyses for the combined association of SCP and ICP on diagnosed dementia in SHARE].

## Data Availability

The data that support the findings of this study are available from the website of the China Health and Retirement Longitudinal Study (CHARLS) at http://charls.pku.edu.cn/ and website of the Survey of Health, Ageing, and Retirement in Europe (SHARE) at https://www.share-eric.eu/.

## Abbreviations

ADL: Activities of daily living
ANOVA: Analysis of variance
BMI: Body mass index
CHARLS: China Health and Retirement Longitudinal Study
ECog: Everyday cognition scale
ELSA: English Longitudinal Study of Ageing
GEE: Generalized estimating equation
HR: Hazard ratio
ICP: Interviewer-reported cognitive problems
MCI: Mild cognitive impairment
OR: Odds ratio
SCC: Subjective cognitive complaints
SCD: Subjective cognitive decline
SCP: Self-reported cognitive problems
SHARE: Survey of Health, Ageing, and Retirement in Europe
