# Supplementary material for: Self- and interviewer-reported cognitive problems in relation to cognitive decline and dementia: results from two prospective studies
Source: BMC Med. 2024 Jan 17;22:23. doi: 10.1186/s12916-023-03147-4 (PMC10792911; doi:10.1186/s12916-023-03147-4)
Supplement: Supplementary file 1 — Additional file 1: SMethods, Figures S1-S4, and Tables S1-S8. SMethods. [Supplementary Methods]. Figure S1. [Flow chart of participants selection for analyzing cognitive decline]. Figure S2. [Flow chart of participants selection for analyzing dementia]. Figure S3. [Age-specific and gender-specific prevalence of self-reported cognitive problems (SCP) and interviewer-reported cognitive problems (ICP) in CHARLS (A) and SHARE (B)]. Table S1. [Association of SCP and ICP on cognitive decline among Chinese (N = 10,976) and European (N = 40,499) middle-aged and older participants]. Figure S4. [Combined association of SCP and ICP on cognitive decline in the domains (episodic memory and executive function) among Chinese (N = 10,976) and European (N = 40,499) middle-aged and older participants]. Table S2. [Association of SCP and ICP on dementia among Chinese (N = 8112) and European (N = 44,849) middle-aged and older participants]. Table S3. [Combined association of SCP and ICP on cognitive decline among Chinese (N = 10,976) and European (N = 40,499) middle-aged and older participants (SCP & ICP group as reference)]. Table S4. [Combined association of SCP and ICP on dementia among Chinese (N = 8112) and European (N = 44,849) middle-aged and older participants (SCP & ICP group as reference)]. Table S5. [Sensitivity analyses for the combined association of SCP and ICP on cognitive decline in CHARLS]. Table S6. [Sensitivity analyses for the combined association of SCP and ICP on cognitive decline in SHARE]. Table S7. [Sensitivity analyses for the combined association of SCP and ICP on probable dementia in CHARLS]. Table S8. [Sensitivity analyses for the combined association of SCP and ICP on diagnosed dementia in SHARE]. [file 12916_2023_3147_MOESM1_ESM.pdf]

Additional file 1

SMethods. Supplementary Methods

Study population

Outcomes included cognitive decline and dementia, with diverse exclusion criteria. As for cognitive decline, participants were excluded if they 1) had no data on self-rated memory, interviewer-rated frequency of asking for clarification, or cognitive function at baseline; 2) were younger than 45 years at baseline; 3) had the diagnosis of diseases severely impairing cognition (brain damage, mental retardation, and memory-related disease including dementia in CHARLS; Alzheimer's disease or dementia in SHARE) at baseline; 4) were regarded as suspected dementia (the lowest 5 percentages in cognitive function) at baseline; 5) had no data on cognitive function at any of the follow-up phases. Final population for analyzing cognitive decline comprised 10976 Chinese in CHARLS and 40499 Europeans in SHARE (Figure S1). As for dementia, participants were excluded if they 1) had no data on self-rated memory or interviewer-rated frequency of asking for clarification at baseline; 2) were younger than 45 years at baseline; 3) had the diagnosis of diseases severely impairing cognition at baseline; 4) were regarded as suspected dementia (with diseases severely impairing cognition at the first follow-up) at baseline; 5) had no data on probable dementia at the last follow-up (the only wave with enough variables to define probable dementia) in CHARLS or diagnosed dementia at any of the follow-up phases in SHARE. Final population for analyzing dementia consisted of 8112 Chinese and 44849 Europeans (Figure S2).

Case definition of probable dementia

According to the operational criteria used in the English Longitudinal Study of Ageing (ELSA), objective cognitive function tests, informant-reported cognitive status of respondents, functional status, and diagnosis of Alzheimer's disease were used together to define probable dementia. In the last follow-up of CHARLS (wave 4), three sets of objective cognitive function tests were administered, covering the domains of concentration, memory function, and verbal fluency and executive function. Those with impairment in two or more domains (defined as scores of 1.5 standard deviations below the mean or lower within each education group) were regarded as having cognitive impairment. Informant-reported cognitive status of respondents was obtained from the informant questionnaire on cognitive decline in the elderly (IQCODE). IQCODE asked informants how participants' states of memory, ability to learn new tasks, judgement, and handling of key everyday situations were compared with previous states, with 5-point scales for answers and higher scores indicating worse states. Based on IQCODE, a mean score of 3.6 was also used as the cut-off value for identification of cognitive impairment. Functional status was measured by activity of daily living (ADL) and difficulty in performing any one of ADL was defined as functional impairment. Probable dementia was defined either as a combination of cognitive impairment and functional impairment, or informant-reported diagnosis of Alzheimer's disease.

Supplementary Tables and Figures Legends

Figure S1. Flow chart of participants selection for analyzing cognitive decline.

Figure S2. Flow chart of participants selection for analyzing dementia.

Figure S3. Age-specific and gender-specific prevalence of self-reported cognitive problems (SCP) and interviewer-reported cognitive problems (ICP) in CHARLS (A) and SHARE (B).

Table S1. Association of SCP and ICP on cognitive decline among Chinese (N = 10976) and European (N = 40499) middle-aged and older participants.

Figure S4. Combined association of SCP and ICP on cognitive decline in the domains (episodic memory and executive function) among Chinese (N = 10976) and European (N = 40499) middle-aged and older participants.

Table S2. Association of SCP and ICP on dementia among Chinese (N = 8112) and European (N = 44849) middle-aged and older participants.

Table S3. Combined association of SCP and ICP on cognitive decline among Chinese (N = 10976) and European (N = 40499) middle-aged and older participants (SCP & ICP group as reference).

Table S4. Combined association of SCP and ICP on dementia among Chinese (N = 8112) and European (N = 44849) middle-aged and older participants (SCP & ICP group as reference).

Table S5. Sensitivity analyses for the combined association of SCP and ICP on cognitive decline in CHARLS.

Table S6. Sensitivity analyses for the combined association of SCP and ICP on cognitive decline in SHARE.

Table S7. Sensitivity analyses for the combined association of SCP and ICP on probable dementia in CHARLS.

Table S8. Sensitivity analyses for the combined association of SCP and ICP on diagnosed dementia in SHARE.

Figure S1. Flow chart of participants selection for analyzing cognitive decline.

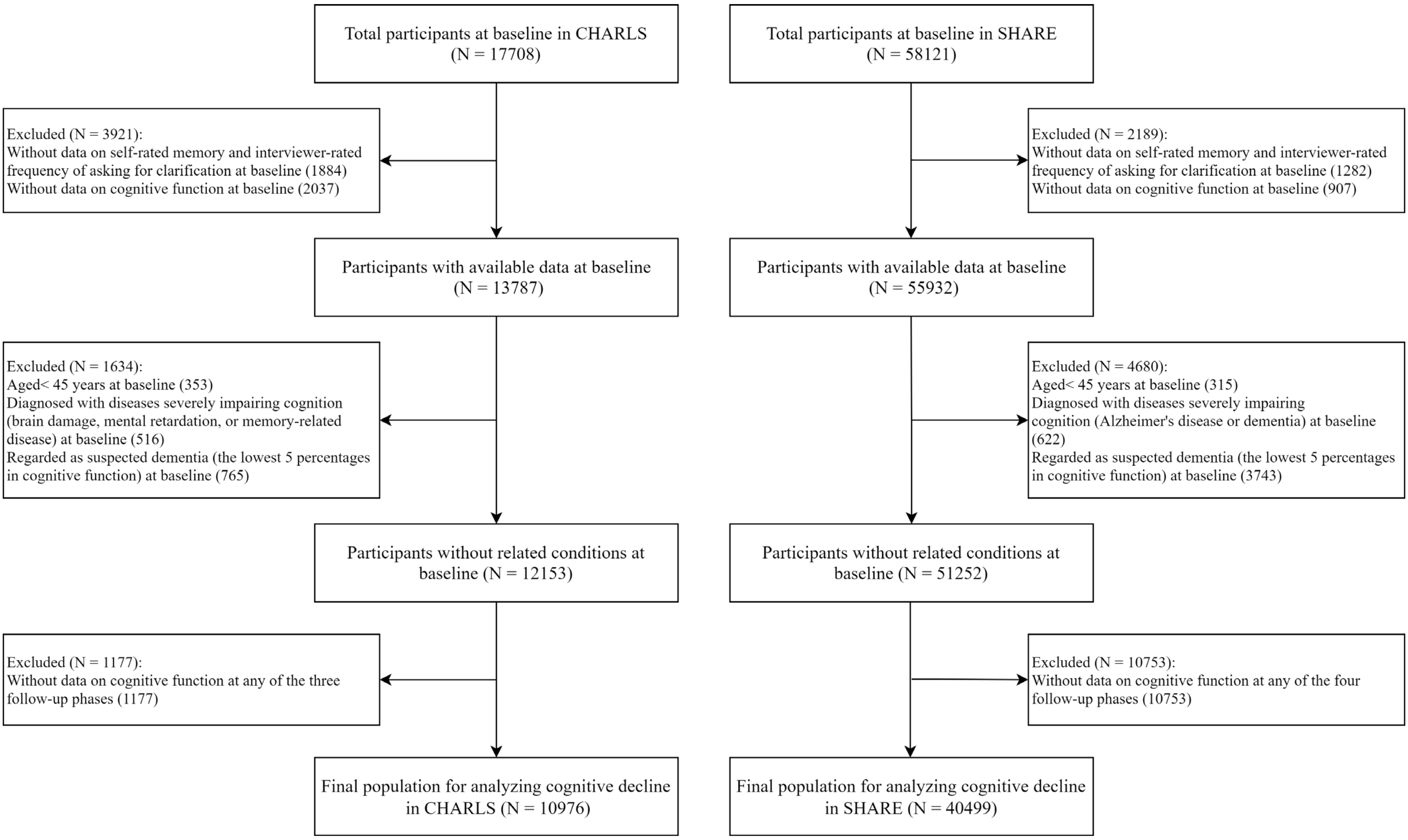

Figure S2. Flow chart of participants selection for analyzing dementia.

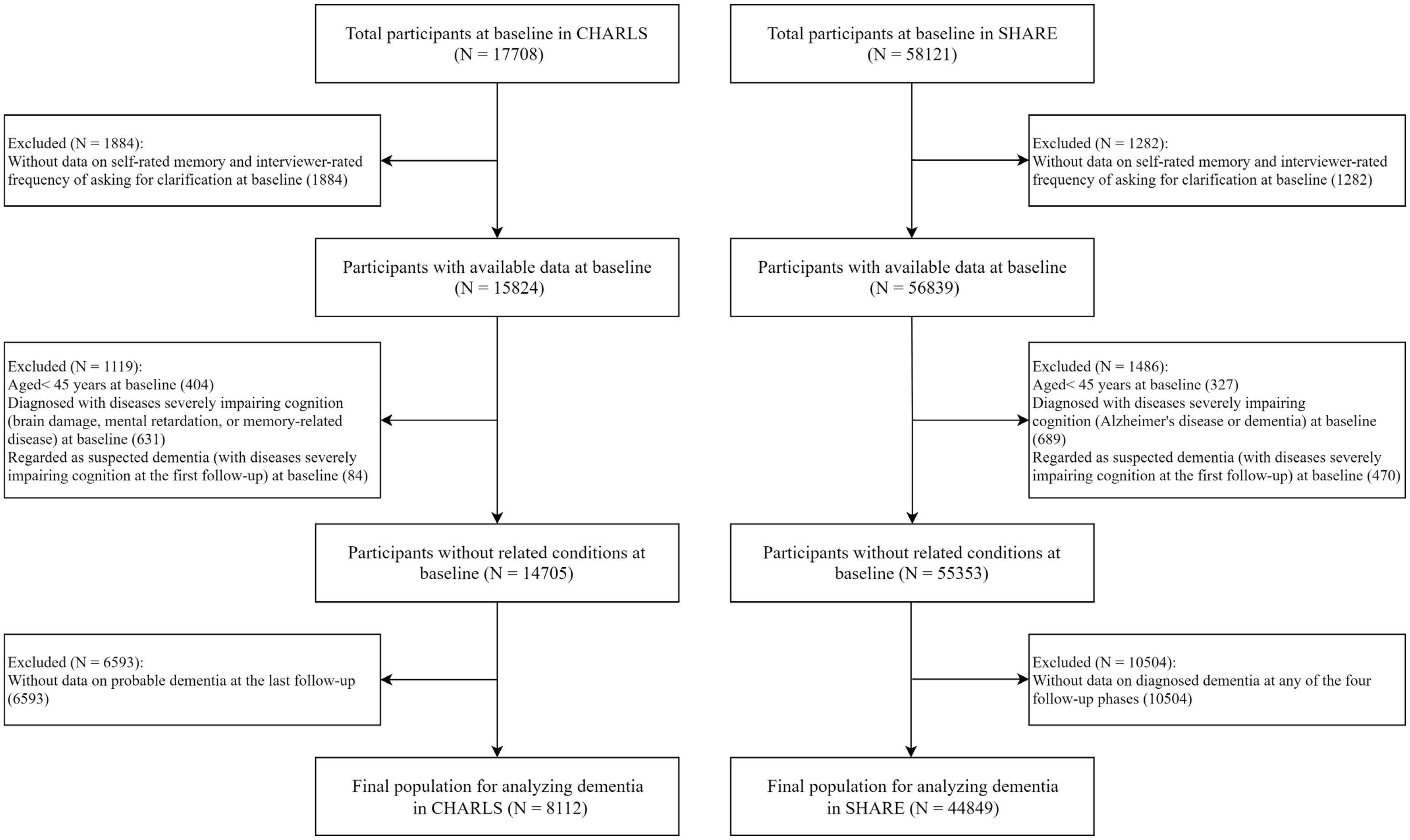

Figure S3. Age-specific and gender-specific prevalence of self-reported cognitive problems (SCP) and interviewer-reported cognitive problems (ICP) in CHARLS (A) and SHARE (B).

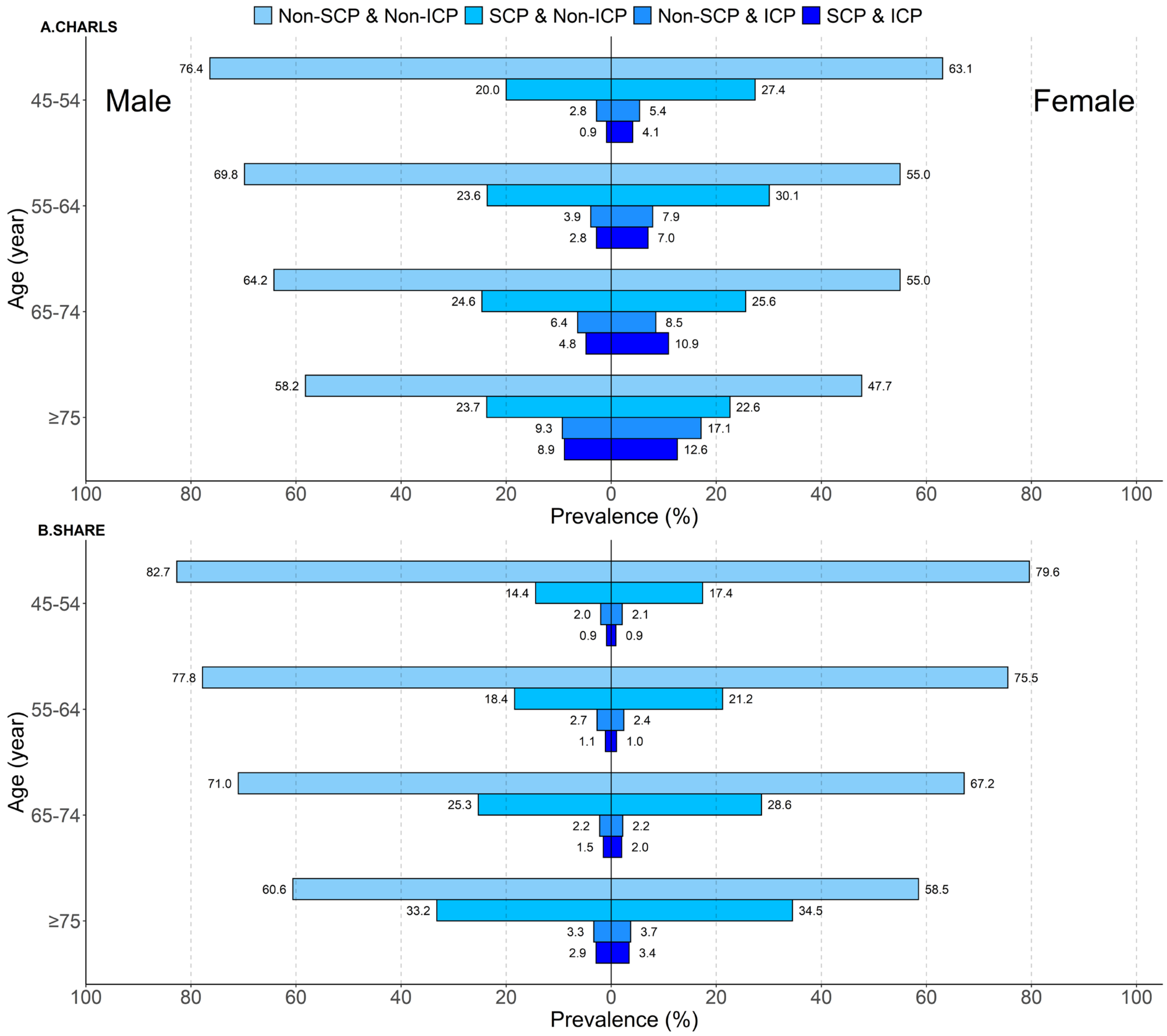

Table S1. Association of SCP and ICP on cognitive decline among Chinese (N = 10976) and European (N = 40499) middle-aged and older participants.

	SCP		ICP	
	**No**	**Yes**	**No**	**Yes**
**China Health and Retirement Longitudinal Study (CHARLS) [a]**				
N	7692	3284	9836	1140
Age-adjusted model	0 (ref.)	-0.009 (-0.021, 0.003)	0 (ref.)	-0.030 (-0.050, -0.010) *
Multivariate model 1 (MV1)	0 (ref.)	-0.008 (-0.019, 0.004)	0 (ref.)	-0.026 (-0.045, -0.006) *
Multivariate model 2 (MV2)	0 (ref.)	-0.008 (-0.019, 0.003)	0 (ref.)	-0.025 (-0.044, -0.006) *
Mutually adjusted model	0 (ref.)	-0.007 (-0.018, 0.005)	0 (ref.)	-0.024 (-0.043, -0.005) *
**Survey of Health, Ageing and Retirement in Europe (SHARE) [b]**				
N	30198	10301	38834	1665
Age-adjusted model	0 (ref.)	-0.002 (-0.007, 0.003)	0 (ref.)	-0.008 (-0.019, 0.004)
Multivariate model 1 (MV1)	0 (ref.)	-0.001 (-0.006, 0.003)	0 (ref.)	-0.006 (-0.018, 0.006)
Multivariate model 2 (MV2)	0 (ref.)	-0.001 (-0.006, 0.004)	0 (ref.)	-0.006 (-0.017, 0.006)
Mutually adjusted model	0 (ref.)	-0.001 (-0.006, 0.004)	0 (ref.)	-0.006 (-0.017, 0.006)
**Pooled Results [c]**				
Multivariate model 2 (MV2)	0 (ref.)	-0.003 (-0.008, 0.003)	0 (ref.)	-0.014 (-0.032, 0.005)
Mutually adjusted model	0 (ref.)	-0.002 (-0.006, 0.003)	0 (ref.)	-0.013 (-0.030, 0.004)

Abbreviations: SCP, self-reported cognitive problems; ICP, interviewer-reported cognitive problems.

Age-adjusted model: adjusted for age, age^2, and follow-up year.

Multivariate model 1 (MV1): CHARLS: further adjusted for gender, residence, marital status, education level, household income, smoking status, drinking status, sleep duration, BMI, depressive symptoms, ADL, and physical activity level; SHARE: further adjusted for gender, residence, marital status, education level, household income, smoking status, drinking status, BMI, depressive symptoms, ADL, vigorous physical activity, moderate physical activity, and country.

Multivariate model 2 (MV2): further adjusted for baseline cognitive function score.

Mutually adjusted model: further adjusted for SCP or ICP (mutually adjusted).

Estimates of the mean differences in cognitive decline across groups were β coefficients of cross-product terms of exposure and follow-up year.

[a]: During an average of 5.4 years of follow-up, the rate of cognitive decline was -0.039 (-0.044, -0.034) z-score/year for Chinese.

[b]: During an average of 5.7 years of follow-up, the rate of cognitive decline was -0.027 (-0.029, -0.025) z-score/year for Europeans.

[c]: Results from the multivariate model 2 (MV2) and mutually adjusted model were combined using the random-effects model.

*: P <0.05.

Figure S4. Combined association of SCP and ICP on cognitive decline in the domains (episodic memory and executive function) among Chinese (N = 10976) and European (N = 40499) middle-aged and older participants.

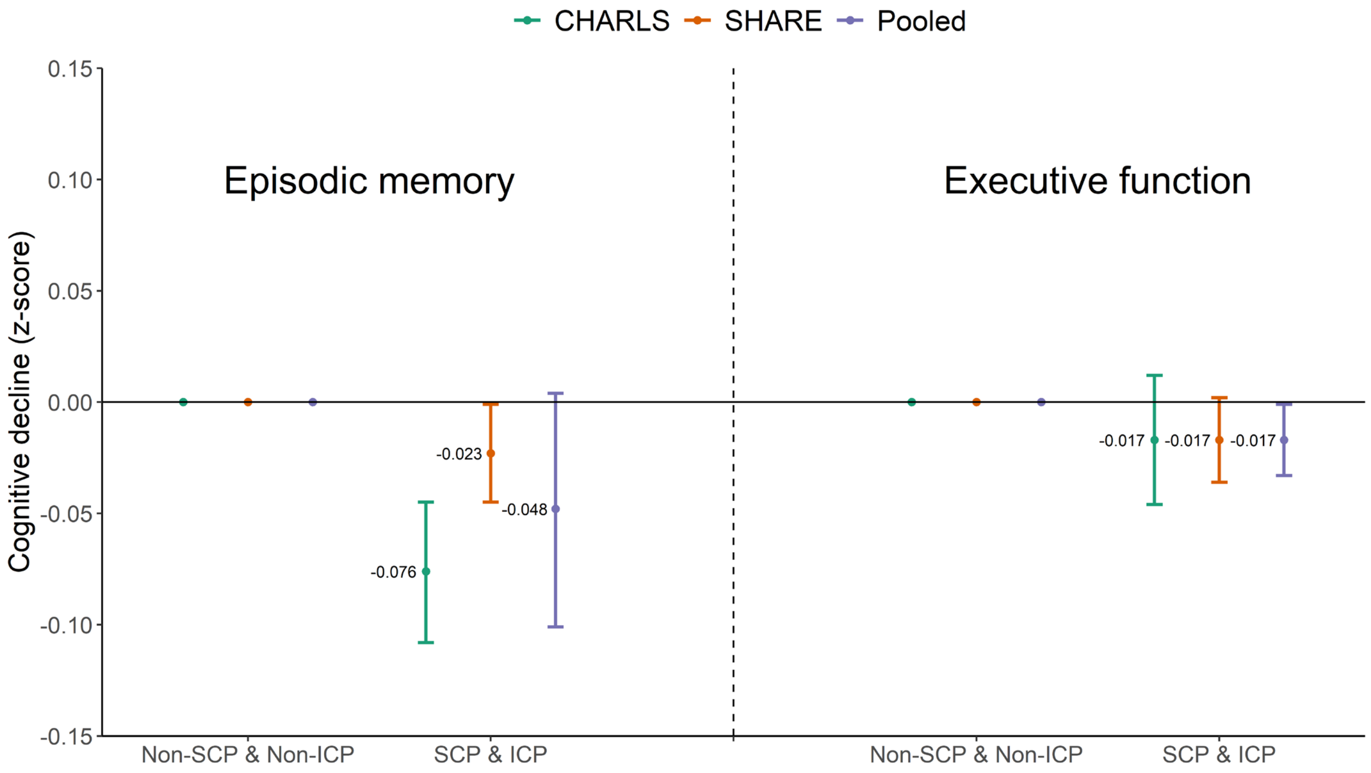

Table S2. Association of SCP and ICP on dementia among Chinese (N = 8112) and European (N = 44849) middle-aged and older participants.

	SCP		ICP	
	No	Yes	No	Yes
**China Health and Retirement Longitudinal Study (CHARLS): Probable dementia, OR (95% CI) [a]**				
N	5127	2985	6771	1341
Cases	525	595	830	290
Age-adjusted model	1 (ref.)	2.11 (1.85, 2.40) *	1 (ref.)	1.63 (1.40, 1.90) *
Multivariate model 1 (MV1)	1 (ref.)	1.49 (1.30, 1.72) *	1 (ref.)	1.23 (1.04, 1.46) *
Mutually adjusted model	1 (ref.)	1.48 (1.28, 1.70) *	1 (ref.)	1.19 (1.01, 1.41) *
**Survey of Health, Ageing and Retirement in Europe (SHARE): Diagnosed dementia, HR (95% CI) [b]**				
N	32535	12314	42411	2438
Cases	701	823	1309	215
Person-years	198242	72536	257212	13566
Incidence rate [d]	353.6	1134.6	508.9	1584.8
Age-adjusted model	1 (ref.)	2.35 (2.12, 2.60) *	1 (ref.)	2.20 (1.90, 2.55) *
Multivariate model 1 (MV1)	1 (ref.)	1.99 (1.78, 2.22) *	1 (ref.)	1.58 (1.36, 1.85) *
Mutually adjusted model	1 (ref.)	1.97 (1.76, 2.20) *	1 (ref.)	1.52 (1.30, 1.77) *
**Pooled Results [c]**				
Multivariate model 1 (MV1)	1 (ref.)	1.73 (1.30, 2.29) *	1 (ref.)	1.40 (1.10, 1.79) *
Mutually adjusted model	1 (ref.)	1.71 (1.29, 2.27) *	1 (ref.)	1.35 (1.06, 1.71) *

Abbreviations: SCP, self-reported cognitive problems; ICP, interviewer-reported cognitive problems.

Age-adjusted model: adjusted for age and age^2.

Multivariate model 1 (MV1): CHARLS: further adjusted for gender, residence, marital status, education level, household income, smoking status, drinking status, sleep duration, BMI, depressive symptoms, ADL, and physical activity level; SHARE: further adjusted for gender, residence, marital status, education level, household income, smoking status, drinking status, BMI, depressive symptoms, ADL, vigorous physical activity, moderate physical activity, and country.

Mutually adjusted model: further adjusted for SCP or ICP (mutually adjusted).

[a]: Odds ratios (OR) were estimated using the logistic regression model.

[b]: Hazard ratios (HR) were estimated using the cox proportional hazards model.

[c]: Results from the multivariate model 1 (MV1) and mutually adjusted model were combined using the random-effects model.

[d]: Incidence per 100,000 person-years.

*: P <0.05.

Table S3. Combined association of SCP and ICP on cognitive decline among Chinese (N = 10976) and European (N = 40499) middle-aged and older participants (SCP & ICP group as reference).

	**Non-SCP & Non-ICP**	**SCP & Non-ICP**	**Non-SCP & ICP**	**SCP & ICP**
**China Health and Retirement Longitudinal Study (CHARLS) [a]**				
N	7064	2772	628	512
Age-adjusted model	0.061 (0.031, 0.092) *	0.059 (0.027, 0.090) *	0.053 (0.014, 0.092) *	0 (ref.)
Multivariate model 1 (MV1)	0.052 (0.023, 0.082) *	0.050 (0.019, 0.081) *	0.044 (0.006, 0.082) *	0 (ref.)
Multivariate model 2 (MV2)	0.051 (0.021, 0.080) *	0.048 (0.017, 0.078) *	0.043 (0.005, 0.080) *	0 (ref.)
**Survey of Health, Ageing and Retirement in Europe (SHARE) [b]**				
N	29177	9657	1021	644
Age-adjusted model	0.028 (0.008, 0.048) *	0.027 (0.007, 0.048) *	0.030 (0.005, 0.054) *	0 (ref.)
Multivariate model 1 (MV1)	0.024 (0.004, 0.044) *	0.024 (0.004, 0.044) *	0.027 (0.003, 0.052) *	0 (ref.)
Multivariate model 2 (MV2)	0.024 (0.004, 0.043) *	0.024 (0.004, 0.044) *	0.027 (0.003, 0.051) *	0 (ref.)
**Pooled Results [c]**				
Multivariate model 2 (MV2)	0.035 (0.009, 0.061) *	0.033 (0.010, 0.056) *	0.032 (0.011, 0.052) *	0 (ref.)

Abbreviations: SCP, self-reported cognitive problems; ICP, interviewer-reported cognitive problems.

Age-adjusted model: adjusted for age, age^2, and follow-up year.

Multivariate model 1 (MV1): CHARLS: further adjusted for gender, residence, marital status, education level, household income, smoking status, drinking status, sleep duration, BMI, depressive symptoms, ADL, and physical activity level; SHARE: further adjusted for gender, residence, marital status, education level, household income, smoking status, drinking status, BMI, depressive symptoms, ADL, vigorous physical activity, moderate physical activity, and country.

Multivariate model 2 (MV2): further adjusted for baseline cognitive function score.

Estimates of the mean differences in cognitive decline across groups were β coefficients of cross-product terms of exposure and follow-up year.

[a]: During an average of 5.4 years of follow-up, the rate of cognitive decline was -0.039 (-0.044, -0.034) z-score/year for Chinese.

[b]: During an average of 5.7 years of follow-up, the rate of cognitive decline was -0.027 (-0.029, -0.025) z-score/year for Europeans.

[c]: Results from the multivariate model 2 (MV2) were combined using the random-effects model.

*: $P < 0.05$.

Table S4. Combined association of SCP and ICP on dementia among Chinese (N = 8112) and European (N = 44849) middle-aged and older participants (SCP & ICP group as reference).

	**Non-SCP & Non-ICP**	**SCP & Non-ICP**	**Non-SCP & ICP**	**SCP & ICP**
**China Health and Retirement Longitudinal Study (CHARLS): Probable dementia, OR (95% CI) [a]**				
N	4495	2276	632	709
Cases	425	405	100	190
Age-adjusted model	0.35 (0.28, 0.42) *	0.70 (0.57, 0.86) *	0.51 (0.39, 0.67) *	1 (ref.)
Multivariate model 1 (MV1)	0.57 (0.45, 0.71) *	0.83 (0.67, 1.03)	0.67 (0.50, 0.89) *	1 (ref.)
**Survey of Health, Ageing and Retirement in Europe (SHARE): Diagnosed dementia, HR (95% CI) [b]**				
N	31259	11152	1276	1162
Cases	637	672	64	151
Person-years	190818	66394	7424	6142
Incidence rate [d]	333.8	1012.1	862.1	2458.5
Age-adjusted model	0.23 (0.20, 0.28) *	0.53 (0.44, 0.63) *	0.46 (0.35, 0.62) *	1 (ref.)
Multivariate model 1 (MV1)	0.34 (0.28, 0.41) *	0.68 (0.56, 0.81) *	0.55 (0.41, 0.74) *	1 (ref.)
**Pooled Results [c]**				
Multivariate model 1 (MV1)	0.44 (0.27, 0.72) *	0.74 (0.61, 0.91) *	0.61 (0.49, 0.74) *	1 (ref.)

Abbreviations: SCP, self-reported cognitive problems; ICP, interviewer-reported cognitive problems.

Age-adjusted model: adjusted for age and age^2.

Multivariate model 1 (MV1): CHARLS: further adjusted for gender, residence, marital status, education level, household income, smoking status, drinking status, sleep duration, BMI, depressive symptoms, ADL, and physical activity level; SHARE: further adjusted for gender, residence, marital status, education level, household income, smoking status, drinking status, BMI, depressive symptoms, ADL, vigorous physical activity, moderate physical activity, and country.

[a]: Odds ratios (OR) were estimated using the logistic regression model.

[b]: Hazard ratios (HR) were estimated using the cox proportional hazards model.

[c]: Results from the multivariate model 1 (MV1) were combined using the random-effects model.

[d]: Incidence per 100,000 person-years.

*: P <0.05.

Table S5. Sensitivity analyses for the combined association of SCP and ICP on cognitive decline in CHARLS.

	**Non-SCP & Non-ICP**	**SCP & Non-ICP**	**Non-SCP & ICP**	**SCP & ICP**
**Sensitivity analysis 1: Multiple imputation for missing covariates (N = 10976).**				
N	7064	2772	628	512
Multivariate model 2 (MV2)	0 (ref.)	-0.003 (-0.015, 0.009)	-0.008 (-0.033, 0.017)	-0.051 (-0.080, -0.021) *
**Sensitivity analysis 2: Without excluding the lowest 5 percentages in global cognitive function at baseline (N = 11566).**				
N	7202	2997	703	664
Multivariate model 2 (MV2)	0 (ref.)	-0.003 (-0.014, 0.007)	-0.013 (-0.034, 0.009)	-0.052 (-0.076, -0.028) *
**Sensitivity analysis 3: Excluding the lowest 10 percentages in global cognitive function at baseline (N = 10153).**				
N	6803	2469	523	358
Multivariate model 2 (MV2)	0 (ref.)	-0.001 (-0.015, 0.013)	0.002 (-0.027, 0.030)	-0.065 (-0.102, -0.027) *
**Sensitivity analysis 4: Excluding those self-reporting the diagnosis of brain damage, mental retardation, or memory-related diseases at any of the three follow-up phases (N = 10193).**				
N	6642	2527	576	448
Multivariate model 2 (MV2)	0 (ref.)	-0.003 (-0.015, 0.010)	-0.011 (-0.036, 0.014)	-0.053 (-0.084, -0.021) *
**Sensitivity analysis 5: Excluding those self-reporting the diagnosis of stroke or cancer at baseline (N = 10646).**				
N	6886	2673	605	482
Multivariate model 2 (MV2)	0 (ref.)	-0.002 (-0.014, 0.010)	-0.012 (-0.037, 0.013)	-0.049 (-0.079, -0.019) *
**Sensitivity analysis 6: Additionally adjusting for the history of hypertension, diabetes, heart-related diseases, stroke, and cancer (N = 10976).**				
N	7064	2772	628	512
Multivariate model 3 (MV3)	0 (ref.)	-0.003 (-0.015, 0.009)	-0.008 (-0.033, 0.017)	-0.051 (-0.081, -0.022) *
**Sensitivity analysis 7: Additionally adjusting for self-rated hearing (N = 10974).**				
N	7064	2772	627	511
Multivariate model 4 (MV4)	0 (ref.)	-0.003 (-0.015, 0.009)	-0.008 (-0.033, 0.016)	-0.050 (-0.079, -0.021) *
**Sensitivity analysis 8: Excluding those with self-rated poor hearing at baseline (N = 9783).**				
N	6649	2255	535	344
Multivariate model 2 (MV2)	0 (ref.)	-0.003 (-0.017, 0.010)	-0.015 (-0.042, 0.011)	-0.057 (-0.093, -0.020) *
**Sensitivity analysis 9: Those who rated their current memory as 'fair' or 'poor' were treated as having SCP (N = 10976).**				
N	2089	7747	164	976
Multivariate model 2 (MV2)	0 (ref.)	0.002 (-0.010, 0.014)	-0.008 (-0.057, 0.042)	-0.026 (-0.049, -0.004) *

Abbreviations: SCP, self-reported cognitive problems; ICP, interviewer-reported cognitive problems.

Multivariate model 2 (MV2): adjusted for age, age^2, follow-up year, gender, residence, marital status, education level, household income, smoking status, drinking status, sleep duration, BMI, depressive symptoms, ADL, physical activity level, and baseline cognitive function score.

Multivariate model 3 (MV3): further adjusted for history of hypertension, diabetes, heart-related diseases, stroke, and cancer.

Multivariate model 4 (MV4): further adjusted for hearing.

*: $P < 0.05$.

Table S6. Sensitivity analyses for the combined association of SCP and ICP on cognitive decline in SHARE.

	Non-SCP & Non-ICP	SCP & Non-ICP	Non-SCP & ICP	SCP & ICP
**Sensitivity analysis 1: Multiple imputation for missing covariates (N = 40499).**				
N	29177	9657	1021	644
Multivariate model 2 (MV2)	0 (ref.)	0.0002 (-0.005, 0.005)	0.004 (-0.010, 0.018)	-0.023 (-0.043, -0.004) *
**Sensitivity analysis 2: Without excluding the lowest 5 percentages in global cognitive function at baseline (N = 42756).**				
N	29949	10613	1177	1017
Multivariate model 2 (MV2)	0 (ref.)	0.001 (-0.003, 0.005)	0.003 (-0.009, 0.016)	-0.011 (-0.026, 0.003)
**Sensitivity analysis 3: Excluding the lowest 10 percentages in global cognitive function at baseline (N = 38460).**				
N	28209	8848	913	490
Multivariate model 2 (MV2)	0 (ref.)	-0.0002 (-0.006, 0.005)	-0.0004 (-0.016, 0.015)	-0.025 (-0.047, -0.003) *
**Sensitivity analysis 4: Excluding those self-reporting the diagnosis of Alzheimer's disease or dementia at any of the four follow-up phases (N = 38505).**				
N	28035	8937	948	585
Multivariate model 2 (MV2)	0 (ref.)	0.001 (-0.004, 0.006)	0.003 (-0.012, 0.018)	-0.024 (-0.046, -0.003) *
**Sensitivity analysis 5: Excluding those self-reporting the diagnosis of stroke or cancer at baseline (N = 36170).**				
N	26415	8268	935	552
Multivariate model 2 (MV2)	0 (ref.)	0.001 (-0.004, 0.006)	0.008 (-0.007, 0.023)	-0.028 (-0.050, -0.006) *
**Sensitivity analysis 6: Additionally adjusting for the history of hypertension, diabetes, heart-related diseases, stroke, and cancer (N = 40499).**				
N	29177	9657	1021	644
Multivariate model 3 (MV3)	0 (ref.)	0.0002 (-0.005, 0.005)	0.004 (-0.010, 0.018)	-0.023 (-0.043, -0.004) *
**Sensitivity analysis 7: Additionally adjusting for self-rated hearing (N = 40498).**				
N	29176	9657	1021	644
Multivariate model 4 (MV4)	0 (ref.)	0.0002 (-0.005, 0.005)	0.004 (-0.011, 0.018)	-0.024 (-0.043, -0.004) *
**Sensitivity analysis 8: Excluding those with self-rated poor hearing at baseline (N = 39239).**				
N	28618	9071	975	575
Multivariate model 2 (MV2)	0 (ref.)	0.0001 (-0.005, 0.005)	0.002 (-0.012, 0.016)	-0.027 (-0.046, -0.007) *
**Sensitivity analysis 9: Those who rated their current memory as 'poor' were treated as having SCP (N = 40499).**				
N	37569	1265	1536	129
Multivariate model 2 (MV2)	0 (ref.)	0.006 (-0.006, 0.018)	-0.005 (-0.017, 0.007)	-0.021 (-0.071, 0.029)

Abbreviations: SCP, self-reported cognitive problems; ICP, interviewer-reported cognitive problems.

Multivariate model 2 (MV2): adjusted for age, age^2, follow-up year, gender, residence, marital status, education level, household income, smoking status, drinking status, BMI, depressive symptoms, ADL, vigorous physical activity, moderate physical activity, country, and baseline cognitive function score.

Multivariate model 3 (MV3): further adjusted for history of hypertension, diabetes, heart-related diseases, stroke, and cancer.

Multivariate model 4 (MV4): further adjusted for hearing.

*: P <0.05.

Table S7. Sensitivity analyses for the combined association of SCP and ICP on probable dementia in CHARLS.

	Non-SCP & Non-ICP	SCP & Non-ICP	Non-SCP & ICP	SCP & ICP
**Sensitivity analysis 1: Multiple imputation for missing covariates (N = 8112).**				
N	4495	2276	632	709
Multivariate model 1 (MV1)	1 (ref.)	1.45 (1.24, 1.71) *	1.16 (0.90, 1.50)	1.74 (1.39, 2.17) *
**Sensitivity analysis 2: Without excluding those self-reporting the diagnosis of brain damage, mental retardation, or memory-related diseases in the first 2 years of follow-up (N = 8151).**				
N	4513	2287	638	713
Multivariate model 1 (MV1)	1 (ref.)	1.47 (1.25, 1.72) *	1.17 (0.91, 1.51)	1.78 (1.43, 2.21) *
**Sensitivity analysis 3: Excluding those self-reporting the diagnosis of stroke or cancer at baseline (N = 7850).**				
N	4373	2196	608	673
Multivariate model 1 (MV1)	1 (ref.)	1.46 (1.24, 1.72) *	1.12 (0.86, 1.46)	1.76 (1.40, 2.21) *
**Sensitivity analysis 4: Additionally adjusting for the history of hypertension, diabetes, heart-related diseases, stroke, and cancer (N = 8112).**				
N	4495	2276	632	709
Multivariate model 2 (MV2)	1 (ref.)	1.45 (1.23, 1.70) *	1.19 (0.92, 1.54)	1.76 (1.41, 2.19) *
**Sensitivity analysis 5: Additionally adjusting for baseline cognitive function (N = 6424).**				
N	3977	1679	409	359
Multivariate model 3 (MV3)	1 (ref.)	1.27 (1.05, 1.54) *	1.22 (0.89, 1.67)	1.49 (1.10, 2.02) *
**Sensitivity analysis 6: Additionally adjusting for self-rated hearing (N = 8108).**				
N	4495	2275	632	706
Multivariate model 4 (MV4)	1 (ref.)	1.38 (1.18, 1.63) *	1.13 (0.87, 1.46)	1.60 (1.28, 2.01) *
**Sensitivity analysis 7: Excluding those with self-rated poor hearing at baseline (N = 6901).**				
N	4157	1789	508	447
Multivariate model 1 (MV1)	1 (ref.)	1.41 (1.18, 1.69) *	1.14 (0.85, 1.53)	1.92 (1.46, 2.51) *
**Sensitivity analysis 8: Those who rated their current memory as 'fair' or 'poor' were treated as having SCP (N = 8112).**				
N	1296	5475	168	1173
Multivariate model 1 (MV1)	1 (ref.)	1.27 (1.02, 1.59) *	1.28 (0.79, 2.07)	1.55 (1.19, 2.02) *

Abbreviations: SCP, self-reported cognitive problems; ICP, interviewer-reported cognitive problems.

Multivariate model 1 (MV1): adjusted for age, age^2, gender, residence, marital status, education level, household income, smoking status, drinking status, sleep duration, BMI, depressive symptoms, ADL, and physical activity level.

Multivariate model 2 (MV2): further adjusted for history of hypertension, diabetes, heart-related diseases, stroke, and cancer.

Multivariate model 3 (MV3): further adjusted for baseline cognitive function.

Multivariate model 4 (MV4): further adjusted for hearing.

Odds ratios (OR) for probable dementia were estimated using the logistic regression model.

*: P <0.05.

Table S8. Sensitivity analyses for the combined association of SCP and ICP on diagnosed dementia in SHARE.

	Non-SCP & Non-ICP	SCP & Non-ICP	Non-SCP & ICP	SCP & ICP
**Sensitivity analysis 1: Multiple imputation for missing covariates (N = 44849).**				
N	31259	11152	1276	1162
Multivariate model 1 (MV1)	1 (ref.)	1.98 (1.76, 2.23) *	1.62 (1.25, 2.10) *	2.92 (2.39, 3.56) *
**Sensitivity analysis 2: Without excluding those self-reporting the diagnosis of Alzheimer's disease or dementia in the first 2 years of follow-up (N = 45319).**				
N	31391	11415	1296	1217
Multivariate model 1 (MV1)	1 (ref.)	2.26 (2.04, 2.51) *	1.71 (1.36, 2.16) *	3.03 (2.55, 3.60) *
**Sensitivity analysis 3: Excluding those self-reporting the diagnosis of stroke or cancer at baseline (N = 39928).**				
N	28300	9499	1163	966
Multivariate model 1 (MV1)	1 (ref.)	1.92 (1.69, 2.19) *	1.50 (1.12, 2.00) *	2.96 (2.37, 3.68) *
**Sensitivity analysis 4: Additionally adjusting for the history of hypertension, diabetes, heart-related diseases, stroke, and cancer (N = 44849).**				
N	31259	11152	1276	1162
Multivariate model 2 (MV2)	1 (ref.)	1.96 (1.74, 2.21) *	1.60 (1.23, 2.08) *	2.87 (2.36, 3.50) *
**Sensitivity analysis 5: Additionally adjusting for baseline cognitive function (N = 40249).**				
N	29089	9509	1013	638
Multivariate model 3 (MV3)	1 (ref.)	1.85 (1.62, 2.11) *	1.40 (1.00, 1.96)	1.97 (1.44, 2.69) *
**Sensitivity analysis 6: Additionally adjusting for self-rated hearing (N = 44848).**				
N	31258	11152	1276	1162
Multivariate model 4 (MV4)	1 (ref.)	1.97 (1.75, 2.22) *	1.59 (1.23, 2.07) *	2.89 (2.36, 3.53) *
**Sensitivity analysis 7: Excluding those with self-rated poor hearing at baseline (N = 43277).**				
N	30639	10418	1202	1018
Multivariate model 1 (MV1)	1 (ref.)	1.91 (1.69, 2.16) *	1.63 (1.24, 2.15) *	3.07 (2.49, 3.79) *
**Sensitivity analysis 8: Those who rated their current memory as 'poor' were treated as having SCP (N = 44849).**				
N	40788	1623	2084	354
Multivariate model 1 (MV1)	1 (ref.)	2.01 (1.69, 2.38) *	1.58 (1.33, 1.89) *	2.64 (2.02, 3.46) *

Abbreviations: SCP, self-reported cognitive problems; ICP, interviewer-reported cognitive problems.

Multivariate model 1 (MV1): adjusted for age, age^2, gender, residence, marital status, education level, household income, smoking status, drinking status, BMI, depressive symptoms, ADL, vigorous physical activity, moderate physical activity, and country.

Multivariate model 2 (MV2): further adjusted for history of hypertension, diabetes, heart-related diseases, stroke, and cancer.

Multivariate model 3 (MV3): further adjusted for baseline cognitive function.

Multivariate model 4 (MV4): further adjusted for hearing.

Hazard ratios (HR) for diagnosed dementia were estimated using the cox proportional hazards model.

*: P <0.05.
